# *Chlamydia psittaci* infection increases mortality of avian influenza virus H9N2 by suppressing host immune response

**DOI:** 10.1038/srep29421

**Published:** 2016-07-11

**Authors:** Jun Chu, Qiang Zhang, Tianyuan Zhang, Er Han, Peng Zhao, Ahrar Khan, Cheng He, Yongzheng Wu

**Affiliations:** 1 Key Lab of Animal Epidemiology and Zoonosis of Ministry of Agriculture, College of Veterinary Medicine, China Agricultural University, Beijing 100193, China; 2 Faculty of Veterinary Medicine, University of Agriculture, Faisalabad 38040, Pakistan; 3 Unit of Cellular Biology & Microbial Infection, CNRS UMR3691, Institut Pasteur, 75015 Paris, France; 4 Shandong Binzhou Animal Science and Veterinary Medicine Academy, Binzhou 256600, China

## Abstract

Avian influenza virus subtype H9N2 (H9N2) and *Chlamydia psittaci* (*C. psittaci*) are frequently isolated in chickens with respiratory disease. However, their roles in co-infection remain unclear. We tested the hypothesis that C. psittaci enhances H9N2 infection through suppression of host immunity. Thus, 10-day-old SPF chickens were inoculated intra-tracheally with a high or low virulence *C. psittaci* strain, and were simultaneously vaccinated against *Newcastle disease virus* (NDV). Significant decreases in body weight, NDV antibodies and immune organ indices occurred in birds with the virulent *C. psittaci* infection, while the ratio of CD4+/CD8+ T cells increased significantly compared to that of the lower virulence strain. A second group of birds were inoculated with *C. psittaci* and H9N2 simultaneously (*C. psittaci*+H9N2), *C. psittaci* 3 days prior to H9N2 (*C. psittaci/*H9N2), or 3 days after H9N2 (H9N2/*C. psittaci*), *C. psittaci* or H9N2 alone. Survival rates were 65%, 80% and 90% in the *C. psittaci*/H9N2, *C. psittaci*+H9N2 and H9N2/*C. psittaci* groups, respectively and respiratory clinical signs, lower expression of pro-inflammatory cytokines and higher pathogen loads were found in both *C. psittaci*/H9N2 and *C. psittaci*+H9N2 groups. Hence, virulent *C. psittaci* infection suppresses immune response by inhibiting humoral responses and altering Th1/Th2 balance, increasing mortality in H9N2 infected birds.

*Chlamydia psittaci* (*C. psittaci*) is an obligate intracellular respiratory pathogen that has a biphasic life cycle within a non-acidified vacuole inclusion^1^. *C. psittaci* can cause psittacosis/ornithosis in poultry and pet birds. Moreover, it is also a human pathogen causing atypical pneumonia after zoonotic transmission. Recent research has reported human pneumonia of varying severity associated with *C. psittaci* infection^2^,^3^. The aforementioned reports indicate that *C. psittaci* infections are highly prevalent and severe respiratory outbreaks in birds and humans have highlighted the importance of *C. psittaci*, although it is frequently neglected as an etiological agent^4^,^5^. A previous study showed that *avian pneumovirus* (APV) infection during the acute phase of a *C. psittaci* infection aggravated the severity of clinical signs, macroscopic lesions, pharyngeal APV excretion and histological tracheae lesions in broiler turkeys^6^. *C. psittaci* and Escherichia coli (*E. coli)* co-infection also exacerbated clinical disease in turkeys^7^. A recent survey indicated *C. psittaci* always preceded *Ornithobacterium rhinotracheale* (ORT) clinical infection in Belgian broilers and high maternal anti-*C. psittaci* antibodies were detected in 1-day-old broilers in the presence of viable *C. psittaci*^8^.

Besides *C. psittaci*, avian influenza H9N2 subtype virus is another major threat to the poultry industry in China due to the limited protection provided by the available inactivated vaccines^9^. Unfortunately, an effective culling strategy is not widely implemented in developing countries during outbreaks of avian H9N2 influenza, so no effective strategy exists for controlling the disease. H9N2 virus infection leads to respiratory distress and is known to contribute to the pathology of other respiratory pathogens in the poultry industry, such as *C. psittaci*^10^,^11^,^12^.

Although *C. psittaci* and H9N2 have been isolated and reported previously^11^, the pathogenic mechanism of co-infection is unclear. We postulated that *C. psittaci* might enhance H9N2 infection through suppression of host immunity. The objective of present study is to reveal the roles of *C. psittaci* and H9N2 in respiratory diseases and study the mechanism of potential immune suppression induced by *C. psittaci* infection.

## Results

### *C. psittaci* infection reduces body weight, immune organ index, NDV-specific antibody level and spleen lymphocytes subsets

**Figure 1:**
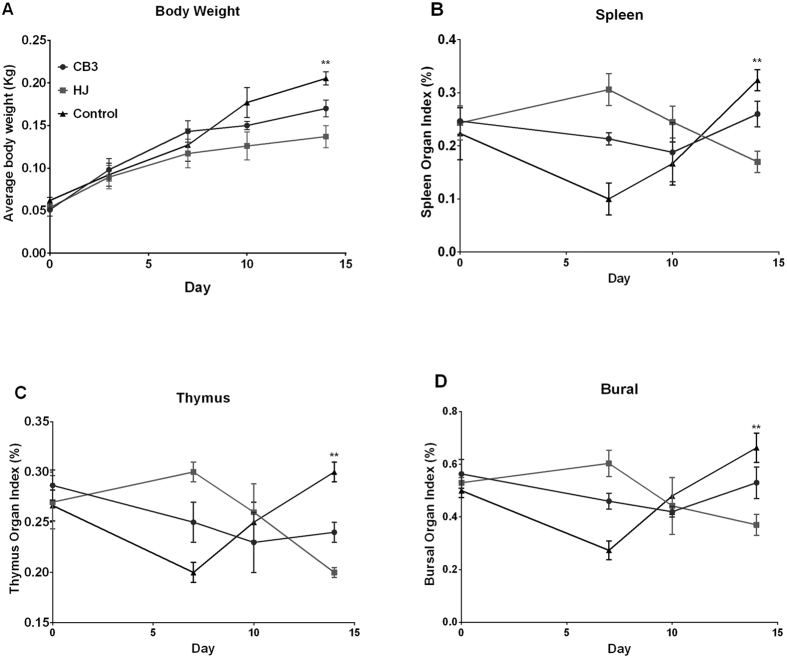
Average body weight and immune organ indices post inoculation with high or low virulence *C. psittaci*. (**A**) Average body weights were significantly reduced in the HJ group compared with the control group on day 14 p.i. (*P* < *0.01*). (**B–D**) Significant increases of spleen organ index, thymus organ index and bursal organ index were found in the HJ group on day 7 p.i., while the above indices were decreased significantly on day 14 compared with the control group (*P* < *0.01*).

Relative average body weight gain was reduced significantly in the high virulence *C. psittaci* HJ strain group (HJ group) in comparison with the low virulence *C. psittaci* CB3 group (*P* < *0.01*) and control group (*P* < *0.01*) on day 10 p.i. (Fig. 1A). With respect to immune organ indices on day 7 p.i., spleen index, thymus index and bursal index were increased significantly in the HJ group compared to the CB3 group (*P* < *0.01*) and the control group (*P* < *0.01*). These indices decreased gradually with no significant differences on day 10 p.i. Subsequently, spleen index, thymus index and bursa index decreased significantly in the HJ group compared to those of the CB3 group (*P* < *0.01*) and control group (*P* < *0.01*) on day 10 p.i. (Fig. 1B–D).

**Figure 2:**
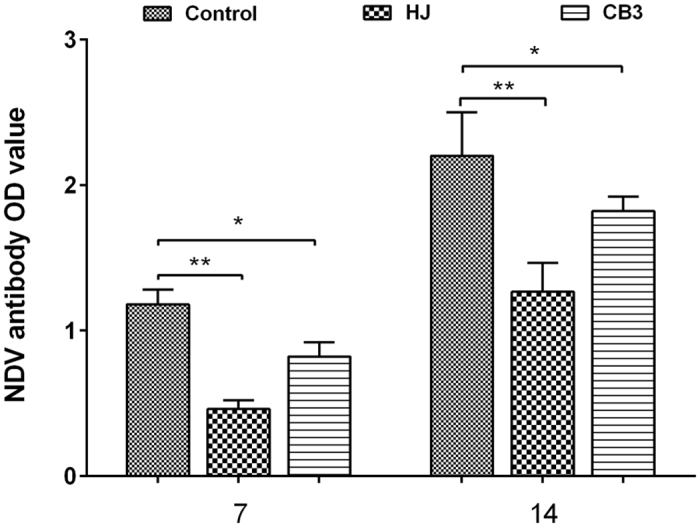
Effect of *C. psittaci* infection on NDV-specific antibodies. NDV-specific antibodies were reduced in the HJ group (*P* < *0.01*) and CB3 group (*P* < *0.05*) in comparison with the control group on day 7 and day 14 p.i. (**P < 0.01) when compared with the control group, *P < 0.05 when compared with the control group).

**Figure 3:**
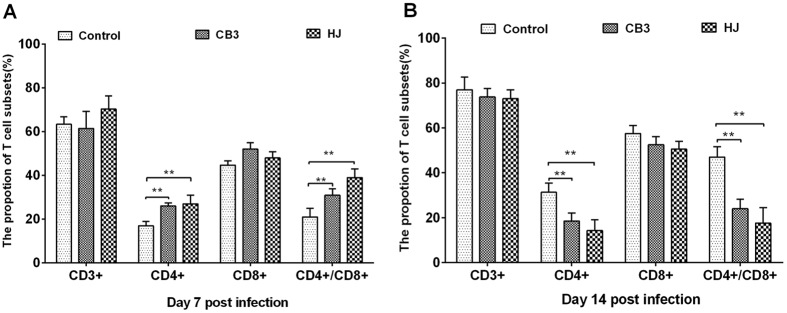
T lymphocyte subsets post inoculation with *C. psittaci.* Proportions of T lymphocyte subsets and the ratio of CD4+/CD8+ were determined by flow cytometry. (**A**) On day 7 p.i. both the CD4+ proportion of T cells and the ratio of CD4+/CD8+ T cells in the HJ group were higher than the CB3 group and control group (*P* < *0.01*). (**B**) On day 14 p.i. both the CD4+ proportion and the ratio of CD4+/CD8+ T cells in the HJ group and the CB3 group were reduced significantly compared with the control group (*P* < *0.01*).

Significant decreases in NDV-specific antibody levels were detected on both day 7 and 14 in the HJ group and CB3 group compared to the control group (Fig. 2). The mean anti-NDV antibody level in HJ group was almost 30% lower when compared with the control group and a significant decrease was also found in the HJ group in comparison with that of the CB3 group at two-time points (*P* < *0.05*) (Fig. 2). Total T cell numbers (CD3+) were comparable among HJ group, CB3 group and the control group on day 7 and day 14 (Fig. 3). Significant increases in the CD4+ (*P* < *0.01*) and CD4+/CD8+ ratio(*P* < *0.01*) were found in the *C. psittaci* HJ group compared to that of *C. psittaci* CB3 group on day 7 (Fig. 3A). By day 14 both the CD4+ cell proportion and the CD4+/CD8+ ratio were significantly reduced (*P* < *0.01*) in the HJ group and the CB3 group compared with those of the control group. Moreover, a significant decrease was found in the HJ group compared to the CB3 group (*P* < *0.01*) (Fig. 3B).

### Co-infection with *C. psittaci* and H9N2 aggravates mortality and multi-organ lesions

**Figure 4:**
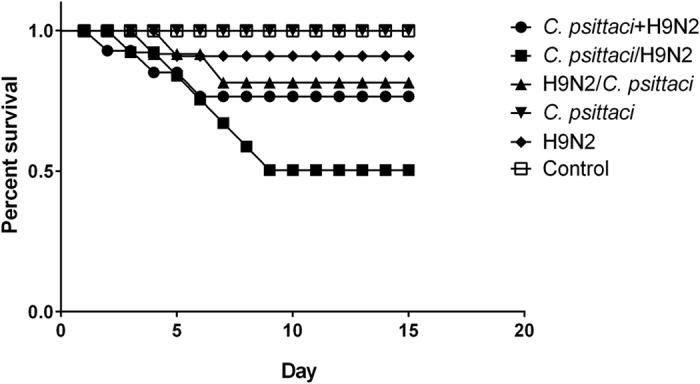
Effect of the co-infection on survival rate. Lower survival rates were found in the *C. psittaci*/H9N2 group (65.0%) and *C. psittaci*+H9N2 group (80.0%) compared with the single agent infection groups (*P* < *0.01*). while 100% survival rate was found in the *C. psittaci* group.

Post infection with *C. psittaci* and H9N2, the infected birds displayed ruffled feathers and poor appetite. Afterwards, 9 out of 15 chickens developed open-mouth breathing in the *C. psittaci*/H9N2 group while 6 out of 15 birds developed breathing difficulty in the *C. psittaci*+H9N2 group. The diseased birds exhibited severe anorexia and began dying on day 2, and mortality peaked on day 8. The survival rate was 65%, 80% and 90%, respectively in the *C. psittaci*/H9N2 group, *C. psittaci*+H9N2 group, and H9N2/*C. psittaci* group. In contrast, all birds inoculated with *C. psittaci* alone survived but exhibited typical breathing difficulty from day 3 to day 7. On the other hand, birds infected with H9N2 alone showed signs of respiratory disease for the first 3 days and recovered thereafter. No deaths occurred in the *C. psittaci* group or H9N2 group alone during the observation period (Fig. 4).

**Figure 5:**
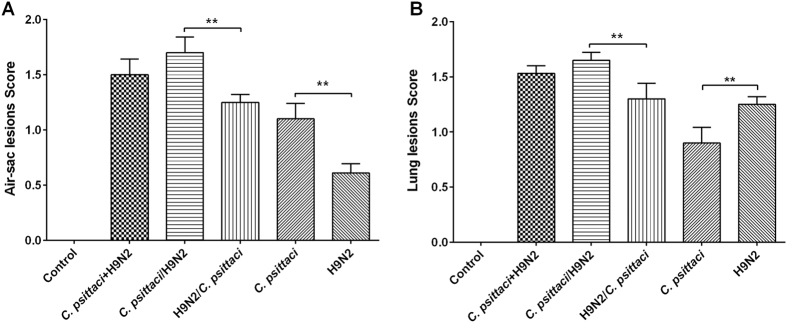
Effect of co-infection with *C. psittaci* and AIV H9N2 on targeted organ lesions. **(A)** A significant increase in air sac lesions was found in the *C. psittaci*/H9N2 group compared to the H9N2/*C. psittaci* group, and in the *C. psittaci* group compared with the H9N2 group on day 14 p.i. (*P* < *0.01*). (**B**) Lung lesions were found significantly increased in the *C. psittaci*/H9N2 group compared to the H9N2/*C. psittaci* group, and in the H9N2 group compared to the *C. psittaci* group (*P* < *0.01*).

On day 14 p.i. significantly more severe air sac lesions were found in the co-infection groups compared with the single infection groups and control group. In addition, significant lesions developed in the *C. psittaci* group compared with the H9N2 group (*P* < *0.01*) (Fig. 5A). As for lung lesions, significantly more lesions developed in the *C. psittaci*/H9N2 group compared with the H9N2/*C. psittaci* group, or *C. psittaci* group or H9N2 group (*P* < *0.01*). However, no statistical difference was found between the *C. psittaci*/H9N2 group and the *C. psittaci*+H9N2 group (Fig. 5B). Significantly more lung lesions were found in the H9N2 groups than in the *C. psittaci* group (*P*  < *0.01*).

On day 4 post infection the indices of bursa, spleen and thymus were significantly increased in the *C. psittaci*/H9N2 group and *C. psittaci*+H9N2 group in comparison with the control group *(P* < *0.01).* Post infection on day 14, the immune organ index was significantly reduced both in the *C. psittaci*+H9N2 group and *C. psittaci*/H9N2 group in comparison with the *C. psittaci* group or H9N2 group (*P* < *0.01*). Post infection on day 7, bursa index, thymus index and spleen index were significantly increased in both the *C. psittaci*/H9N2 group and *C. psittaci*+H9N2 group in comparison with the control group (*P* < *0.05*). On day 14 p.i. the immune organ indices were significantly reduced in both the *C. psittaci*+H9N2 group and *C. psittac*i/H9N2 group in comparison with the *C. psittaci* group or H9N2 group (*P* < *0.05*).

### Co-infection with *C. psittaci* and H9N2 decreased cytokine mRNA expressions and virus/bacterial clearance in the lungs

**Figure 6:**
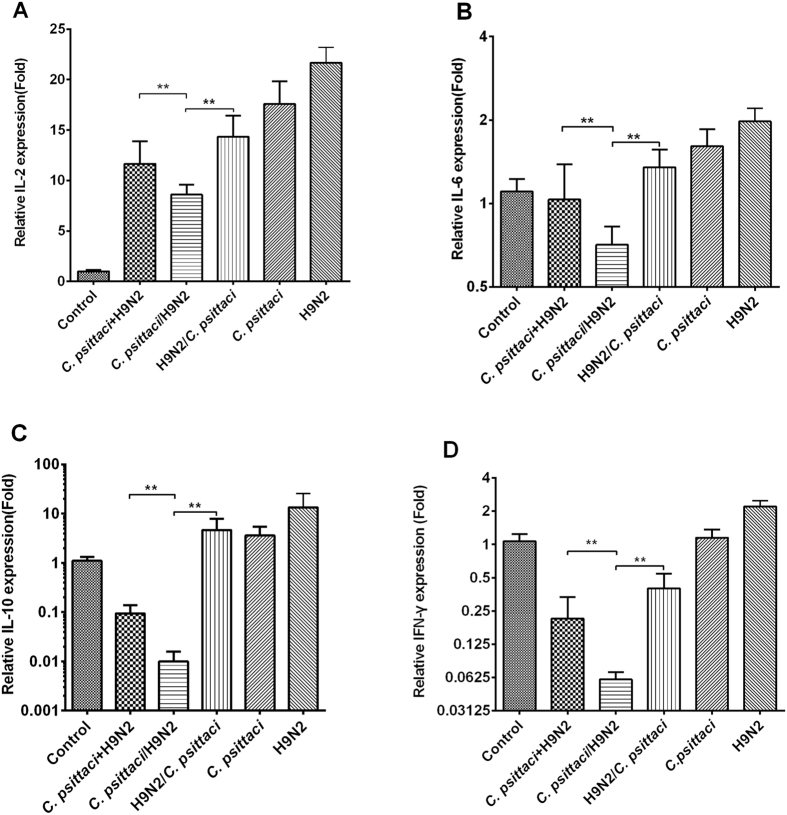
Effect of co-infection on cytokine expressions. The mRNA expressions of IL-2, IL-6, IL-10 and IFN-γ were found to be significant decrease in the *C. psittaci*/H9N2 group compared to those of the *C. psittaci*+H9N2, H9N2/*C. psittaci, C. psittaci*. or H9N2 groups on day 14 p.i. (*P* < *0.01*) (**A–D**).

Expression of IL-2, IL-6, IL-10 and IFN-γ decreased significantly in the *C. psittaci*+H9N2 group and *C. psittaci*/H9N2 group compared to that of the H9N2/*C. psittaci* group or *C. psittaci* alone group on day 14. Moreover, a significant decrease was found in the *C. psittaci*/H9N2 group compared to that of the *C. psittaci*+H9N2 group or H9N2/*C. psittaci* group (*P* < *0.01*) (Fig. 6).

**Figure 7:**
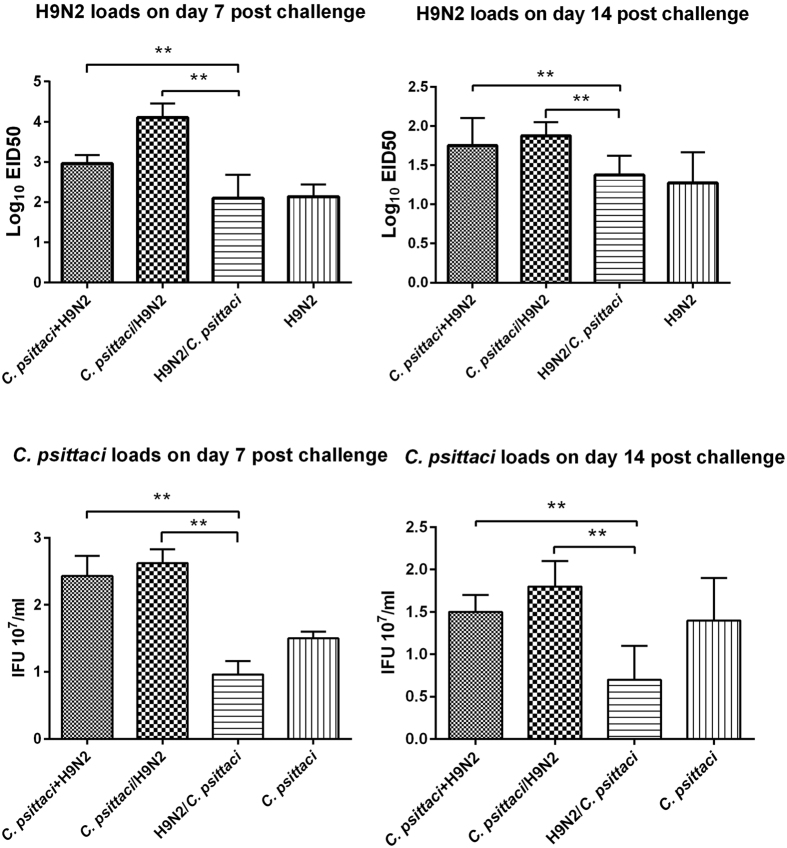
Pathogen loads on day 7 and day 14, respectively post challenge in chicken lungs. Significantly higher H9N2 and *C. psittaci* loads were detected in the *C. psittaci*+H9N2 group and *C. psittaci*/H9N2 group in comparison with those of the H9N2/*C. psittaci* group or *C. psittaci* group both on day 7 (*P* < *0.01*) and 14 (*P* < *0.01*).

Besides inflammatory cytokines, we also determined the viral/bacterial burdens. Higher H9N2 virus loads were determined in the *C. psittaci*+H9N2 group and *C. psittaci*/H9N2 group compared to the H9N2/C. *psittaci* or H9N2 group both on day 7 and day 14 *(P* < *0.01)*. The highest bird flu virus load was observed in the lungs of the *C. psittaci*/H9N2 group on day 7 p.i.*(P* < *0.01)*. Afterwards, high virus loads were maintained in the *C. psittaci*+H9N2 and *C. psittaci*/H9N2 group, but no significant difference was found between these two groups on day 14 (Fig. 7). The post infection chlamydial loads were statistically greater in the *C. psittaci*+H9N2 group and *C. psittaci*/H9N2 group compared to those of the H9N2/*C. psittaci* and *C. psittaci* group on both day 7 and day 14 post infection (*P* < *0.01*) (Fig. 7).

## Discussion

In the present study, chickens inoculated with the high virulence *C. psittaci* strain exhibited immune suppression, characterized by lower relative body weight gain, degeneration of immune indices, and decreasing CD4+/CD8+ ratio and NDV-specific antibody levels. Higher mortality, severe respiratory distress and higher levels of virus shedding were observed in the *C. psittaci*/H9N2 group compared with the H9N2/*C. psittaci* group or H9N2 group. All the above data support our hypothesis that a primary *C. psittaci* infection will aggravate the infection of avian influenza virus subtype H9N2 by suppressing immune organs and adaptive immune responses in chickens.

In our pilot study, we found hemorrhagic lesions in both bursa and thymus after infection with a mildly pathogenic *C. psittaci* strain^13^, similar to the pathology described in *Infectious Bursal Diseases* (IBD)^14^. In the current study, chickens inoculated with *C. psittaci* had lower bursa index and thymus index compared to the control group. Anti-NDV antibody titers decreased on both day 7 and 14 following infection with *C. psittaci*. Our results suggest that the highly virulent *C. psittaci* strain contributes to the impairment of the immune response by damaging bursa and thymus organs. With respect to lymphoid cell populations later in the infection, CD4+ subsets decreased significantly in the highly virulent *C. psittaci* HJ group. Our study provides background for a report that a highly virulent *C. psittaci* strain led to higher mortality and more severe clinical signs and lesions compared with a low virulence strain^15^.

In previous reports the characteristic necropsy lesions of birds infected with *C. psittac*i included an enlarged spleen, liver and fibrinous exudate on respiratory, peritoneal, and pericardial surfaces. Clinical signs in birds infected with a low virulence strain were anorexic and had loose green droppings^16^. Regarding zoonotic implications^17^, severe cases of human psittacosis have been reported^18^,^19^,^20^,^21^. However, *C. psittaci*-associated immune suppression is unknown in both birds and human beings.

In the present study the administration of *C. psittaci* 3 days prior to, or together with, H9N2 contributed to the mortality as well as the lesion severity in target organs (air sacs and lungs). The survival rates were 65% and 80% in the *C. psittaci*/H9N2 and *C. psittaci*+H9N2 groups respectively, compared with 100% survival rate in the *C. psittaci* single infection group and the high survival rate in the H9N2 group. Clinical signs and mortality in the *C. psittaci*/H9N2 and *C. psittaci*+H9N2 groups were similar to those reported in the broiler industry, which ranges from 25% to 35% dead birds during seasonal outbreaks of airsacculitis^11^. However, the exact mechanism of synergism between these two pathogens in the course of co-infection is unknown. Here we speculate that *C. psittaci* infection activates host defense suppression by initiating immune organ damage, altering production of inflammatory mediators and modifying organ specific defense mechanisms. In this way, these interactions might augment viral adherence, making birds susceptible to H9N2 and other infections. Indeed, a recent study demonstrated that both APV and *C. psittaci* were identified in Belgian broilers with respiratory disease. During acute *C. psittaci* infection, APV infection might play an exacerbating role in respiratory disease complex of turkeys^22^. Moreover, co-infection of *C. psittaci* with fowl poxvirus has been reported in commercial laying hens with 20% mortality. The results of electron microscopic examination suggested that it was easier for Chlamydiae to penetrate cell membranes weakened by primary poxvirus infection^23^. However, in the current study high mortality rates were observed when the *C. psittaci* infection occurred prior to or together with the H9N2 infection.

In addition to animal cases, the dual infection with *C. psittaci* and Sjögren syndrome (SS) in humans supports our hypothesis. *C. psittaci* was detected at higher frequency in salivary gland mucosa-associated lymphoid tissue (MALT) lymphoma patients (6/18, 33.3%), compared with myoepithelial sialoadenitis (MESA) (3/20, 15%) or patients without lymphoproliferative disorders ((2/36, 5.6%). *C. psittaci* infection could be involved in a fraction of patients with SS developing lymphoma by suppressing host innate defenses^24^.

Currently, AIV H9N2 is still subject to seasonal outbreaks in China and poses a huge threat to the poultry industry. Lack of fundamental investigations and a basic understanding of the disease pathogenesis hinders the control of H9N2-associated respiratory diseases and its interactions with other pathogens. In the present study, SPF chickens inoculated with H9N2 alone did not exhibit significant mortality, while mild hemorrhagic pneumonia was the typical lesion compared with severe fibrinous exudate and pneumonia seen in *C. psittaci* infected birds. Prior, or simultaneous, infection with *C. psittaci* might facilitate the continued survival of H9N2 by reducing INF-γ secretion and thereby contribute to the high mortality compared to the H9N2/*C. psittaci* or H9N2 group. The Th1 response is important in the host defense against bacterial or viral infection^25^. Highly virulent *C. psittaci* infection appears to impair the Th1/Th2 balance and AIV H9N2 might benefit from the persistent infection, with higher levels of virus shedding and more severe histopathological changes resulting in long-term damage to the chickens. This might account for the persistent airsacculitis and pneumonia in broiler farms. This is the first time that co-infection with *C. psittaci* and H9N2 induce immune suppression in chickens. Further study is needed to check whether *C. psittaci*-mediated immune suppression results in the impairment of vaccine approaches.

Viral-bacterial co-infection often increases disease severity in both humans and animals. Understanding the mechanisms and effects of co-infection improves the understanding of their zoonotic implications. Recently co-infection of a novel adenovirus and *C. psittaci* was reported in affected parrots in Hong Kong^26^. The role of viral-bacterial co-infection in animal-to-human transmission of infectious agents has not received sufficient attention and should be emphasized in the investigation of disease outbreaks in human and animals.

Our findings differ from those reports in which a viral infection dominates the primary infection, followed by a secondary bacterial infection. We hypothesise that *C. psittaci* infection plays a major role in respiratory diseases compared to H9N2.

In conclusion, our study is the first report that a primary *C. psittaci* or a long-term latent infection may lead to immune suppression *in vivo* which will increase susceptibility to other pathogens, such as H9N2 and APV. It suggests that we should consider a primary infection by *C. psittaci.* in any respiratory disease and should eradicate *C. psittaci* during treatment of avian respiratory disease.

## Materials and Methods

### Animals

Specified pathogen free (SPF) birds aged 10 days purchased from Weitong Merial Laboratory Animal Co., Ltd, Beijing and kept at Animal Experimental Center, China Agricultural University (Beijing, China). All animals were maintained in strict accordance with the Regulations for the Administration of Affairs Concerning Experimental Animals of the State Council of the People’s Republic of China. The protocols were approved by the Committee on Experimental Animal Management of the China Agricultural University.

### Pathogen origin

Low virulence strain CB3 and high virulence HJ strain of *C. psittaci* were isolated from free-living birds^27^ and diseased pigeons^28^. AIV H9N2/chicken/Shandong/2011 was isolated from broilers as described previously^11^. The attenuated vaccine against Newcastle Diseases Virus (NDV) was purchased from Ceva-Huadou Co.Ltd, Beijing, China.

### Effect of *C. psittaci* infection on organ lesions and immune responses

Forty-eight 10-day-old SPF chickens were divided into 3 groups, 16 birds per group. Group 1 birds received 1 × 10^8.5^ IFU of *C. psittaci* CB3 strain by intratracheal (it) inoculation and Group 2 birds were inoculated with the same dose of the high virulence *C. psittaci* HJ strain, while control group birds received the same volume of physiological saline (it). At the same time all the above birds received intranasal inoculation (i.n.) of attenuated vaccines against NDV, one dose per chicken. Blood samples were collected by venipuncture on day 7 and day 14 post infection (p.i.) and NDV-specific antibodies measured using commercial kits (IDEXX, USA). Meanwhile, body weight gains were detected on the above two days. Thymus, bursa and spleen organs were removed to determine organ index following humane euthanasia. Immune organ indices were calculated according to the formula: index (mg/g) = (weight of thymus, spleen or bursa of Fabricius)/body weight.

### Effect of *C. psittaci* and H9N2 co-infection on organ lesions and mortality

Ninety-six, 10-day-old SPF chickens were divided into 6 groups, 16 birds per group. The *C. psittaci*+H9N2 group animals were inoculated simultaneously with 1 × 10^8.5^ IFU *C. psittaci* HJ strain (it) and with 100 ELD_50_ H9N2 virus (in). The *C. psittaci*/H9N2 group received 1 × 10^8.5^ IFU *C. psittaci* HJ strain (it) then 100 ELD_50_ H9N2 (in) in 0.2 ml three days later. The H9N2/*C. psittaci* group animals were inoculated with 100 ELD_50_ H9N2 (in) and then inoculated (it) with 1 × 10^8.5^ IFU *C. psittaci* HJ strain three days later. The *C. psittaci* group birds were given 1 × 10^8.5^ IFU *C. psittaci* HJ strain (it) while the H9N2 group birds were administered 100 ELD_50_ H9N2 (in). Control group birds received sterile physiological saline as a negative control. Birds were monitored daily and the immune organ indices determined on day 0, day 7, day 10 and day 14. Four birds were euthanized per time point in each group.

Air sac lesions were determined as previously described. Briefly, air sac lesions were divided into five grades, 0 score— normal, clean, thin and transparent; 1 score— slightly thickened and slightly turbid, or individual local white exudate; 2 score—grayish white exudate in a few areas of the air sac, moderate sac thickness; 3 score—majority of the air sacs are fully covered with yellow white caseous exudate and thickening of air sacs is obvious; 4 score—serious air sac lesions with white thick exudate on thoracic cavity and abdominal cavity^29^. Lung lesions were determined as previously described^30^. Briefly, the degree of oedema was semi-quantitatively graded in H&E stained lung sections: grade 0 = none; grade 1 = slight oedema of the alveolar walls; grade 2 = moderate oedematous thickening of alveolar walls with occasional alveoli containing coagulated oedema fluid; grade 3 = extensive occurrence of alveolar and interstitial oedema.

Spleen lymphocytes were collected for the measurement of CD3+, CD4+ and CD8+ cells as previously described^14^. Briefly, 1 × 10^6^ spleen lymphocytes were incubated with anti-chicken CD3-SPRD, anti-chicken CD4-FITC and anti-chicken CD8-RPE (Southern Biotech, USA) at 4 °C for 30 min. Subsequently, lymphocytes were washed 3 times with phosphate buffered saline containing 1% fetal bovine serum, then re-suspended and analyzed by FacsCalibur and CellQuest software (Becton Dickinson, USA). Viable lymphocytes were calculated based on forward and sideward scatter characteristics, and 10,000 events analyzed for positive staining with SPRD, FITC and RPE antibody.

### Quantitative real-time reverse transcription PCR

After euthanasia, lungs were lavaged three times with 1mL of HBSS and centrifuged. The bronchus alveolar lavage fluid (BALF) supernatants were discarded and the cell pellets used for further assays. Total RNA was extracted from BALF cell pellet lymphocytes by applying Trizol (TransGen Biotech, Beijing, China) and subsequently treating with DNA-free kit to filter DNA contamination. First strand cDNA was transcribed using TransGen Reverse Transcription kit (TransGen Biotech, Beijing, China) according to the manufacturer’s protocols. Relative quantification of IL-2, IL-6, IL-10, IFN-γ was performed using SYBR Green PCR Master Mix kit (Takara, Dalian, China), The 2^−ΔΔCt^ method was used to calculate relative gene expression levels. Gene expression was assayed quantitatively and standardized to the level of a housekeeping gene (GAPDH) to obtain a RNA ratio in order to establish relevant change in RNA expression^31^.

### Pathogen loads

Whole lungs were collected to assess pathogen loads. Briefly, 100 μg samples were immersed in 900 ul PBS containing antibiotics (Gentamicin 2000 IU/ml and Streptomycin 2000 IU/ml) and then homogenized. The homogenate was maintained at 4 °C for 40 min and then centrifuged at 2000 rpm/min for 5 min to obtain the supernatant. Each supernatant was divided into two samples and maintained at −80 °C until used. One sample was used for H9N2 determination by inoculating into 10-day-old SPF embryonated eggs as described previously^11^. The second sample was used to infect BGMK cells to make the determination of inclusion body units (IFUs).

### Statistical analysis

Data are expressed as means ± SEM (standard error of the mean) and analyzed using STATISTCA v.7 (Stat Soft) software. Nonparametric analysis and Mann–Whitney U tests performed for comparison between groups and the data presented as median values. Multiple group analysis included the multiple comparison correction (Bonferroni). Statistically significant differences were judged as *p* < *0.05*.

## Additional Information

**How to cite this article**: Chu, J. *et al*. *Chlamydia psittaci* infection increases mortality of avian influenza virus H9N2 by suppressing host immune response. *Sci. Rep.* **6**, 29421; doi: 10.1038/srep29421 (2016).
